# FAP and FAPI-PET/CT in Malignant and Non-Malignant Diseases: A Perfect Symbiosis?

**DOI:** 10.3390/cancers13194946

**Published:** 2021-09-30

**Authors:** Katharina Dendl, Stefan A. Koerber, Clemens Kratochwil, Jens Cardinale, Rebecca Finck, Mardjan Dabir, Emil Novruzov, Tadashi Watabe, Vasko Kramer, Peter L. Choyke, Uwe Haberkorn, Frederik L. Giesel

**Affiliations:** 1 Department of Nuclear Medicine, Heidelberg University Hospital, 69120 Heidelberg, Germany; clemens.kratochwil@med.uni-heidelberg.de (C.K.); jens.cardinale@med.uni-heidelberg.de (J.C.); rebecca.finck@med.uni-heidelberg.de (R.F.); uwe.haberkorn@med.uni-heidelberg.de (U.H.); frederik.giesel@med.uni-duesseldorf.de (F.L.G.); 2 Department of Nuclear Medicine, Düsseldorf University Hospital, 40225 Düsseldorf, Germany; mardja.dabir@med.uni-duesseldorf.de (M.D.); emil.novruzov@med.uni-duesseldorf.de (E.N.); 3 Department of Radiation Oncology, Heidelberg University Hospital, 69120 Heidelberg, Germany; stefan.koerber@med.uni-heidelberg.de; 4 Heidelberg Institute of Radiation Oncology (HIRO), 69120 Heidelberg, Germany; 5 National Center for Tumor Diseases (NCT), Heidelberg University Hospital, 69120 Heidelberg, Germany; 6 Department of Nuclear Medicine and Tracer Kinetics, Osaka University Graduate School of Medicine, Osaka 565-0871, Japan; tadashi.watabe@med.uni-osaka.de; 7 Positronpharma SA, Santiago 7500921, Chile; vkramer@positronpharma.cl; 8 Center of Nuclear Medicine, PositronMed, Santiago 7501068, Chile; 9 Molecular Imaging Program, Center for Cancer Research, National Cancer Institute, National Institutes of Health, Bethesda, MD 20892-1088, USA; pchoyke@mail.nih.gov; 10 Clinical Cooperation Unit Nuclear Medicine, German Cancer Research Center (DKFZ), 69120 Heidelberg, Germany; 11 Translational Lung Research Center Heidelberg, German Center for Lung Research DZL, 69120 Heidelberg, Germany

**Keywords:** FAPI-PET/CT, FAP, CAFs, various malignancies, benign diseases, fibroblast

## Abstract

**Simple Summary:**

FAPI represents a novel class of radiotracers demonstrating promising results in terms of a high uptake in concordance with low background noise in several malignancies. Thereby, FAPI-PET/CT achieves sharp contrasts facilitating staging as well as tumor delineation and detection. However, FAP is also overexpressed for several non-oncological reasons allowing for benign indications as well. This review summarizes the current state of oncological and non-oncological FAPI-PET/CT in accordance with FAP in order to highlight future perspectives and identify areas where research is urgently warranted.

**Abstract:**

A fibroblast activation protein (FAP) is an atypical type II transmembrane serine protease with both endopeptidase and post-proline dipeptidyl peptidase activity. FAP is overexpressed in cancer-associated fibroblasts (CAFs), which are found in most epithelial tumors. CAFs have been implicated in promoting tumor cell invasion, angiogenesis and growth and their presence correlates with a poor prognosis. However, FAP can generally be found during the remodeling of the extracellular matrix and therefore can be detected in wound healing and benign diseases. For instance, chronic inflammation, arthritis, fibrosis and ischemic heart tissue after a myocardial infarction are FAP-positive diseases. Therefore, quinoline-based FAP inhibitors (FAPIs) bind with a high affinity not only to tumors but also to a variety of benign pathologic processes. When these inhibitors are radiolabeled with positron emitting radioisotopes, they provide new diagnostic and prognostic tools as well as insights into the role of the microenvironment in a disease. In this respect, they deliver additional information beyond what is afforded by conventional FDG PET scans that typically report on glucose uptake. Thus, FAP ligands are considered to be highly promising novel tracers that offer a new diagnostic and theranostic potential in a variety of diseases.

## 1. Introduction

Fibroblast activation protein (FAP), a membrane-anchored serine protease with dipeptidyl peptidase and endopeptidase activity, is overexpressed by cancer-associated fibroblasts (CAFs) [1]. CAFs alter the tumor microenvironment and enhance protumorigenic effects [2]. FAPI-PET/CT has emerged as a highly promising imaging tool, particularly in conditions where FDG, the standard oncologic PET tracer, is of limited use such as in pancreatic cancer. Moreover, FAPI may have more general advantages over FDG because of its higher target to background ratio, caused by the very low expression of FAP in normal tissue and a rapid elimination from the circulation, which increases the sensitivity. The PET agent, ^68^Ga-FAPI, has already been tested on a variety of tumor entities in humans. ^68^Ga-FAPI has been less extensively studied in inflammation, wound healing and other benign diseases [3,4,5]. In this review, we summarize the current status of FAPI-PET/CT in malignant and non-malignant diseases and highlight particularly interesting opportunities for this new imaging method.

## 2. Tumor Biology

### 2.1. Stroma

Malignant tumors contain various substituents including neoplastic cells as well as non-malignant cells in the tumor microenvironment, termed the tumor stroma. The tumor stroma may represent up to 90% of cells in several cancers and therefore may constitute the predominant component of many tumors such as colon, breast and pancreatic cancer [6]. By the time a tumor reaches a size of 2 mm, the tumor stroma is essential for growth as it supplies the neoplastic cells with nutrients to ensure survival and growth [7,8]. Cancer-associated fibroblasts are joined by endothelial cells, the basement membrane, the extracellular matrix and immune cells in the tumor microenvironment (TME) and act as a supporting cast for tumor growth.

### 2.2. Fibroblasts

Healthy fibroblasts are ubiquitous and are generally biologically quiescent. However, these resting fibroblasts harbor the ability to be activated under various circumstances. This process was first described in connection with wound healing [9] and subsequently at sites of inflammation and tissue fibrosis [10,11,12]. Moreover, activated fibroblasts secrete more extracellular matrices and support a higher cell proliferation [13] compared with fibroblasts in healthy tissues.

Fibroblast activation is enabled in a context-dependent manner including acute wound healing from mechanical trauma, radiation or toxins [12,14,15,16]. Wound healing leads to inflammation and the recruitment of immune cells and fibroblasts [12,17,18] as part of the repair mechanism. After repair, the number of activated fibroblasts rapidly diminishes due to apoptosis and resting fibroblasts return as the predominant type of cell. Reversibility is the determinative element with respect to wound healing, with activated fibroblasts giving way to mature fibroblasts. Fibroblasts may be activated in a number of ways including fibrosis itself, a process referred to as chronic wound healing [19,20,21,22]. Ultimately, fibroblast activation can also be caused by genetically damaged cells that mimic inflamed cells resulting in tumors or “wounds that do not heal” [17] with a positive feedback mechanism whereby more tumor growth results in more activated fibroblasts. Thus, the growth of cancer cells presents recurring tissue damage ensuring a permanent wound healing response leading to stromal or cancer fibrosis [17]. The full mechanism by which fibroblasts are activated and promote tumor growth is not yet known and requires further research [23].

### 2.3. Cancer-Associated Fibroblasts (CAFs)

**Figure 1:**
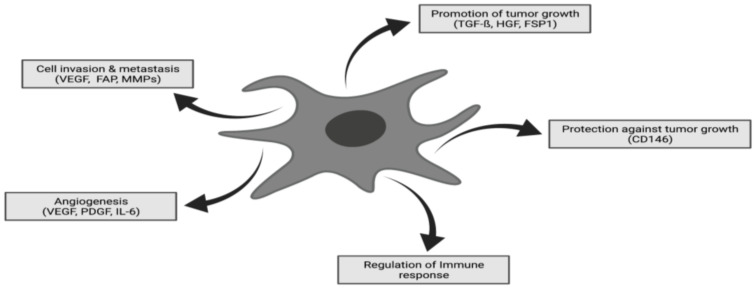
CAFs are in possesion of various influental functions in neoplastic tissue including promotion of tumor growth, cell invasion and metastasis, angiogenesis, regulation of immune response and protection against tumor growth. Thereby, CAFs are representing one of the key players in terms of tumor progression and therapeutic response. Adapted with permission from ref. [33].

**Figure 2:**
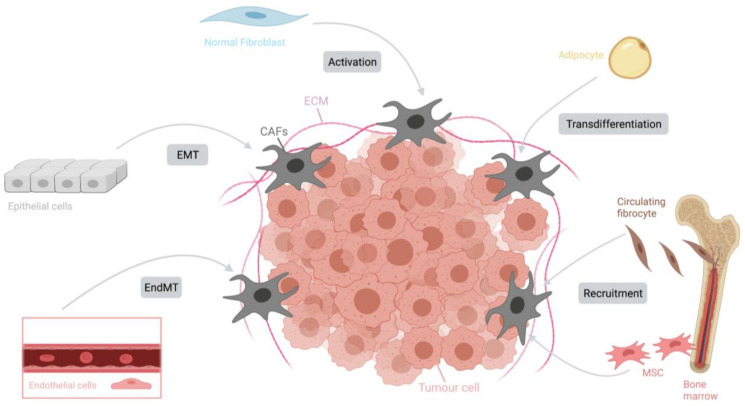
Origins of CAFs. CAFs represent a very heterogenous population deriving from several different cell origins such as normal resident tissue fibroblasts, fibrocytes, mesenchymal cells, epithelial and endothelial cells as well as adipocytes, pericytes and smooth muscle cells. The transformation to CAFs occurs in concordance with distinct mechanisms including activation, transdifferentiation, mesenchymal transition and recruitment. Adapted with permission from ref. [33].

CAFs represent the most abundant TME cells in many cancers. However, CAFs are, in reality, a heterogenous population of cells as both tumor-promoting and tumor-suppressive effects can be observed in CAF-rich tumors [24]. Activated CAFs are characterized by an enhanced proliferation and migration and an increased recruitment. (Figure 1) CAFs can be derived from normal resident tissue fibroblasts [25], fibrocytes originating in the bone marrow [26], mesenchymal cells [27,28], epithelial and endothelial cells undergoing a mesenchymal transition [29,30] and—less commonly—adipocytes, pericytes and smooth muscle cells going through transdifferentiation [20,31] accounting for their variable phenotypes (Figure 2) [32].

Quiescent and activated fibroblasts can be differentiated based on several specific characteristics. Morphological differences can be observed as resting fibroblasts are typically spindle shaped cells [34] whereas activated fibroblasts are cruciform or stellate shaped. Although a definitive cell defined marker of activated CAFs is yet to be agreed upon, Kalluri suggested that fibroblast-specific protein 1 may be the most reliable marker [23,35]. Quiescent fibroblasts, which are metabolically inactive, possess the ability to be activated by a variety of stimuli. For instance, local hypoxia and oxidative stress enhances CAF activation mediated by TGFß [36], PDGF [37] and IL-6 [38]. By an autocrine signaling loop, they provide the activation of more quiescent fibroblasts ensuring growth and the invasion of the tumor. Thus, CAFs co-evolve alongside neoplastic cells and provide continuous support to cancer growth.

### 2.4. Fibroblast Activation Protein

Fibroblast activation protein (FAP) is a serine protease containing both dipeptidyl peptidase (DPP) and endopeptidase activities [39], which differentiates it from other DPP IV family members. FAP dimerizes and glycosylates when activated [40] and is rare in normal adult tissue but can be found in embryogenesis [41]. Thus, activated FAP is almost exclusively found in wound healing and in pathological conditions such as scar formations [42], liver cirrhosis [43], inflammation [44,45] and cancer.

## 3. FAP Expression and FAPI-PET/CT in Non-Oncological Conditions

### 3.1. Liver Fibrosis and Cirrhosis

FAP is associated with scar formations. Thus, upregulation has been observed in fibrotic conditions such as liver fibrosis induced by chronic damage due to alcoholism or viral hepatitis. In this setting, hepatic stellate cells are activated and generate an extracellular matrix conducive to the formation of scars within the liver that express FAP [43,46]. The elevation of FAP levels can correlate with the progression and grade of liver fibrosis in a few cases [43]. Remarkably, a normal FAP expression has been observed after a liver transplant [47]. A case report by Zhao et al. describes a cirrhotic patient presenting multiple liver nodules of an uncertain origin. ^68^Ga-FAPI-PET/CT demonstrated an increased liver uptake due to cirrhosis. Moreover, the nodules showed an even higher uptake but were proven to be benign. Therefore, FAPI-PET/CT may have difficulties in differentiating between malignant and benign nodules in patients with cirrhotic disease [48] A further study presented a significantly elevated hepatic uptake in patients with cirrhosis compared with patients without cirrhosis. On the other hand, FDG-PET/CT showed no significant differences in the background liver activity [49]. Therefore, FAP overexpression in a benign fibrosis may challenge the diagnostic performance of FAPI-PET/CT in further clinical applications. However, a benign accumulation of FAP ligands demonstrate a lower uptake compared with malignant diseases and the distinction of benign and malignant processes is thus feasible.

### 3.2. Crohn’s Disease

Crohn’s disease (CD), an autoimmune condition, results in chronic bowel inflammation and is characterized by intestinal fibrosis and strictures. Chronic inflammation and dysregulated wound healing leads to an intestinal stricture formation. FAP is significantly upregulated in the myofibroblasts within the muscle layer of the strictures but not in myofibroblasts from patients with ulcerative colitis [50]. Furthermore, immunohistochemistry staining proved FAP expression in samples from fibrostenotic areas in patients diagnosed with CD [51]. This research was validated by a report, showing an intense uptake in a patient with CD whereas no elevated accumulation could be observed in ulcerative colitis [52].

### 3.3. Arthritis

Arthritis is defined as any condition resulting in joint dysfunction. The two most frequently occurring types are osteoarthritis, correlating with the proteolytic destruction of joint cartilage, and rheumatoid arthritis, an autoimmune chronic inflammatory condition [53,54]. Rheumatoid arthritis is accompanied by FAP upregulation and thus might present a potential therapeutic target [44]. In osteoarthritis, higher levels of chondrocyte FAP expression were detected by Milner et al. and subsequently validated by comparing the mRNA of collagen from patients with and without osteoarthritis. Moreover, immunohistochemistry revealed FAP expression on the surface of the cartilage and on chondrocyte membranes [55]. These findings are supported by a report of a patient with an intense uptake within the joints who was undergoing a FAPI-PET/CT scan based on a history of shoulder osteoarthritis [56]. Accordingly, inflammatory polyarthritis was accompanied by an intense FAPI uptake in another patient whereas only a slight FDG uptake was seen in the same patient [57].

### 3.4. Cardiovascular Disease

Cardiovascular disease includes such conditions as atherosclerosis and myocardial infarction (MI). FAP is expressed by human aortic smooth muscle cells induced by TNFalpha and is therefore associated with thin-cap human fibroatheromas [45]. In further studies of atherosclerosis-related conditions, reduced levels of FAP in acute coronary syndromes and coronary heart diseases were observed. Patients with an acute coronary syndrome and a decreased FAP expression may exhibit a poorer prognosis [58,59]. However, one study that aimed to investigate FAP expression after MI in rats and human hearts demonstrated FAP upregulation peaking within 7 days after an induced MI; in particular, in the peri-infarct zone. Consistently, in the ischemic tissue of human hearts an increased FAP expression was observed whereas none was observed within normal heart tissues [60]. These results were confirmed by Varasteh et al. who used ^68^Ga-FAPI uptake in rats after a coronary litigation to demonstrate a peak uptake at day 6 post-MI. This study also revealed that most of the uptake was in the border-ischemic area [61]. FAPI accumulated beyond the extent of the infarcted zone, thereby revealing a tendency to overestimate the infarct size [62]. However, this may be caused by a remodeling in the peri-infarct zone as a result of perfusion deficits also in this area. A case report of uptake in the left ventricular myocardium on FAP imaging was attributed to a potential drug-induced cardiotoxicity in an asymptomatic patient. This suggests a role for ^68^Ga-FAPI as an imaging tool for detecting chemotherapy-induced myocardial injuries [63]. In a study of 229 (modeling cohort *n* = 185; confirmatory cohort *n* = 44) patients with a metastatic disease, an association was observed between the cardiovascular risk factors as well as metabolic diseases and increased ventricular FAPI uptake suggesting it may be an early biomarker of a cardiac disease [64]. Another analysis found a correlation between coronary artery diseases, age and the left ventricular ejection fraction with FAPI accumulation [65]. Regarding myocarditis, particularly due to checkpoint inhibitors, FAPI showed an increased accumulation [66]. In summary, FAPI-PET/CT may be a useful imaging tool in cardiovascular diseases and more studies are warranted to assess and validate its use in this setting.

### 3.5. IgG4-Related Disease

IgG4 is a disease affecting multiple organs with fibrosis. A study containing 26 patients with IgG4-related disease demonstrated more multiple affected sites than were evident on the basis of the symptoms. In addition, FAPI revealed a superiority over FDG in detecting such lesions with a higher positivity rate [67]. Moreover, a study including 27 patients with inflammatory, fibrotic and IgG4-related diseases assessed FAPI-PET/CT, FDG-PET/CT, MRI and histopathological evaluations. They determined that FAPI-PET/CT allows for differentiation of inflammatory- or fibrotic-based IgG4-related diseases [68]. This knowledge may affect and lead to changes in the therapeutic approaches as IgG4-related diseases, foremost comprising fibrosis, possibly require distinct treatments. IgG4-related disease can affect the pancreas as well. An analysis of 103 patients undergoing ^68^Ga-FAPI-04 PET/MR showed a focally increased non-malignant uptake in the pancreas in seven cases from causes such as pancreatic pseudocysts, prior pancreatitis and IgG4-related diseases [69].

In a translational exploratory study, Röhrich et al. investigated 15 patients with fibrotic interstitial lung diseases and suspected lung cancer undergoing FAPI-PET/CT scans as well as selective FAP immunohistochemistry. This evaluation revealed promising results encouraging further studies [70].

### 3.6. Benign Tumors

**Figure 3:**
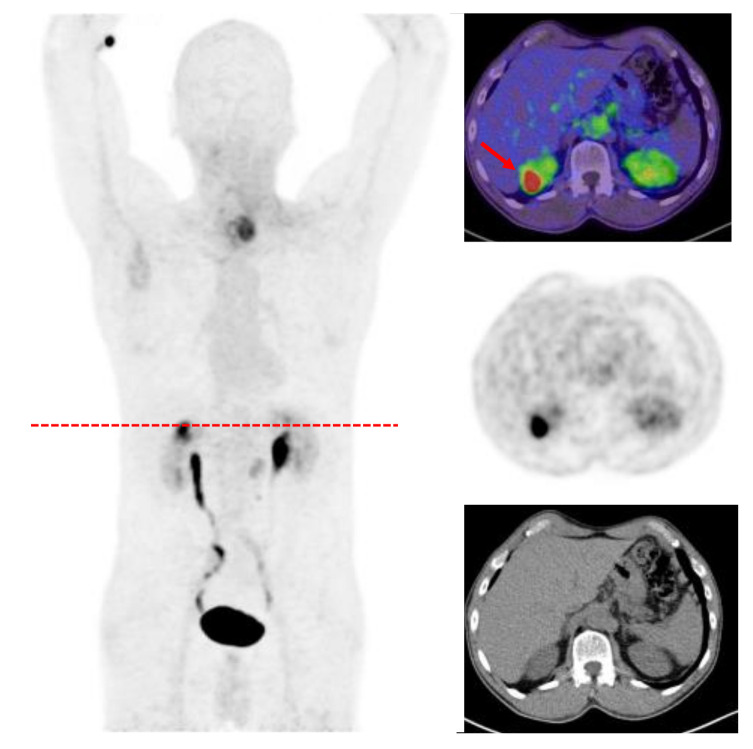
A 57-year-old patient underwent FAPI-PET/CT due to hypopharyngeal cancer and presented an incidental finding of an angiomyolipoma in the upper pol of the right kidney with a SUVmax of 15.9 as well as an accumulation located left paratracheal suspicious for neoplastic tissue (SUVmax 11.5).

Benign tumors with a FAPI uptake have so far been mostly described as incidental findings in case reports. Exemplary is a recurrent angiomyolipoma demonstrating an increased FAPI accumulation possibly due to fibrotic activity in this condition [71] (Figure 3). Another case described an elevated uptake of FAP ligands in a patient with a benign pulmonary solitary fibrous tumor [72]. Zheng et al. characterized benign lesions in 182 patients with various suspected malignancies based on conventional imaging, clinical information and/or a histological confirmation. Out of these 182 patients 146 presented benign lesions (*n* = 360) such as inflammation-based processes, exostoses, hemorrhoids, fractures and hepatic fibroses. However, the SUV metrics demonstrated a lower FAPI uptake in the benign lesions compared with the malignant accumulation [73]. However, it is still too early to describe a role for FAPI imaging in benign tumors and more experience will be gathered as more patients undergo this scan.

### 3.7. Hormone-Responsive Organs

Hormone-responsive organs seem to be associated with an increased uptake on FAPI-PET/CT. In an analysis regarding FAPI uptake in the breast, ovary and uterus, statistically significant differences were seen in the breast and ovaries with a stronger uptake in pre-menopausal patients [74]. Additionally, the uptake might correlate with the menstrual cycle. In a post-partum woman and two women receiving hormonal stimulation, elevated FAP levels and therefore an increased FAPI uptake was observed in the breasts and uterus [75,76,77]. Thus, information regarding the hormonal status of a patient may be relevant to the observed findings.

## 4. Malignant Conditions

**Figure 4:**
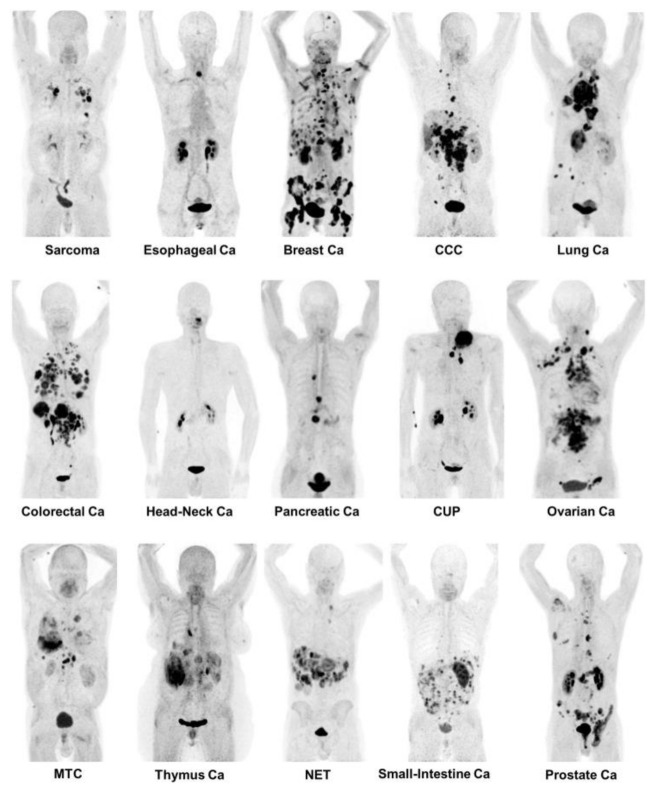
Depiction of Maximum Intensity Projections of several patients undergoing FAPI-PET/CT including a variety of different tumor entities. Adapted with permission from ref. [79].

The overexpression of FAP in many malignancies is now well-known. Therefore, FAPI-PET/CT is a highly promising imaging method in a variety of malignancies [78,79,80] (Figure 4). The exact role of FAP in a tumorigenesis is unknown; however, its expression is related to the tumor proliferation, migration and invasion [46]. To date, the literature suggests that FAP influences angiogenesis, presumably based on its enzymatic activity [46]. Furthermore, FAP seems to be involved in the epithelial-to-mesenchymal transition (EMT), which is eventually required for the development of metastasis by enabling migration and invasion. As FAP is only overexpressed by activated fibroblasts, it is likely that CAFs play a major role in cancer growth.

### 4.1. Brain, Head and Neck Cancer

As mentioned above, the application of glucose-based PET is limited in brain diseases by a high physiologic uptake complicating the detection of the tumor uptake. By contrast, FAPI has a low physiologic background activity in the brain, enabling the detection of small tumors in the brain. Non-malignant lesions and low-grade astrocytoma seem to lack FAP expression. However, in glial tumors, FAP levels correlate with the grade and thus the overexpression is associated with a poor prognosis [81,82,83,84]. Röhrich et al. investigated FAP and FAPI-PET/CT in gliomas in vitro, in vivo and in a clinical study including 18 glioma patients (five IDH-mutant gliomas and 13 IDH-wildtype glioblastomas). The study determined elevated levels of tracer uptake in high-grade gliomas but not in low-grade IDH-mutant gliomas, potentially enabling a non-invasive diagnostic distinction [85]. Furthermore, Windisch et al. evaluated the above-mentioned IDH-wildtype glioblastomas regarding their usefulness for radiotherapy or biopsy planning. Compared with MRI, FAPI-PET/CT scans lead to increased gross tumor volumes (GTVs), thus changing radiation treatment fields [86].

In head and neck cancer, a correlation between FAP and the aggressiveness of these tumors suggests that FAP ligands may be a promising imaging tool. An initial study of 14 patients diagnosed with head and neck cancers showed a high uptake within the malignant lesions and simultaneously a low background activity in the healthy tissue such as the salivary glands. This could lead to an improved target volume delineation compared with FDG PET-CT [87]. Head and neck cancers are usually imaged with ^18^FDG-PET/CT despite the low specificity of this method. Linz et al. evaluated 10 patients with an oral squamous cell carcinoma undergoing both FAPI and FDG-PET/CT. FAPI and FDG presented a similar uptake in the primary tumors as well as a similar sensitivity and specificity in cervical lymph node metastases. The FAP expression of all investigated lesions was histologically confirmed by immunohistochemistry [88]. Additionally, adenoid cystic carcinomas—which are characterized by an epithelial origin, a location in the head and neck area and finally by infiltrative growth—represent a highly interesting target for FAP ligands. However, in a series of 12 patients with adenoid cystic carcinomas, a strong FAPI uptake was established within all patients that correlated with FAP overexpression in the tumor. FAPI-PET/CT thus facilitated staging and radiotherapy planning in comparison with conventional structural imaging such as CT and MRI [89]. The head and neck regions, as well as head and neck cancers in particular, warrant further research as the so-far published results seem to be encouraging but are too provisional to determine the true value of FAPI imaging.

### 4.2. Gastrointestinal Tract Cancer

FAP is highly expressed in gastric cancers [90,91] and FAP levels may be associated with higher grades, peritoneal infiltration and a worse prognosis [92,93]. In an early study of gastric cancer, a higher uptake and sensitivity was observed with ^68^Ga-FAPI-PET/CT over ^18^F-FDG-PET/CT [94]. An analysis by Qin et al. showed a superior performance of FAPI-PET/MR in comparison with FDG-PET/CT for both primary and secondary gastric cancer lesions [95]. FAPI is also more sensitive for peritoneal carcinomatosis, which is otherwise a challenging imaging diagnosis [96]. In a study of 46 patients with peritoneal carcinomatosis due to various cancers, FAPI imaging demonstrated a high sensitivity [96]. These auspicious results warrant further research and suggest a role in the diagnosis of peritoneal carcinomatosis.

In esophageal cancer, Ristau et al. investigated the role of FAPI-PET/CT in 7 patients with esophageal cancer. They demonstrated a strong uptake, high TBRs and, consequently, a precise target volume delineation with FAPI PET/CT resulting in an improved radiotherapeutic management [97]. These findings were subsequently confirmed by Zhao et al. in 21 patients with newly diagnosed esophageal cancer who underwent both ^18^F-FDG and ^68^Ga-FAPI-PET/CT scans. FAPI imaging showed favorable SUV metrics, TBRs and a superior target volume delineation [98].

**Figure 5:**
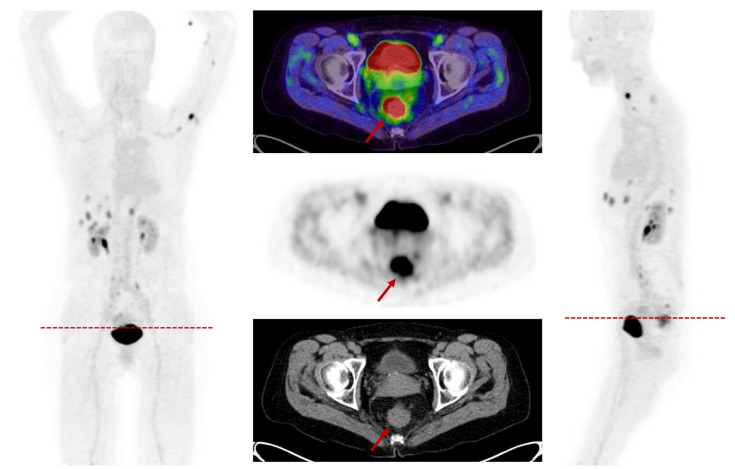
A 57-year-old female patient presenting with newly diagnosed rectal cancer for staging prior to a resection. The FAPI-PET/CT demonstrated a rather strong FAPI accumulation in the rectum with a SUVmax of 15.8. Additionally, the scan showed multiple lymphatic and hepatic metastases.

Colorectal cancer (CRC) is characterized by FAP overexpression within neoplastic cells and the tumor stroma [99] and is a marker seen early in the development of CRC [100]. FAP levels correlate with a poor prognosis and a higher grade, stage and invasion [99,100,101]. Radiotherapy can further increase FAP expression [101]. A first clinical analysis of 28 patients with malignancies of the lower gastrointestinal tract demonstrated high uptakes and TBRs in CRC (*n* = 22) and anal cancer (*n* = 6), which led to clinically relevant changes in the TNM classification and an improved target volume delineation in almost all cases [102] (Figure 5).

A further study investigated the potential of FAP imaging compared with FDG imaging in 35 patients with gastric, duodenal and colorectal cancer. FAPI imaging demonstrated higher uptakes and a higher sensitivity in primary and secondary colorectal cancer compared with FDG [94]. These results suggest that FAPI-PET/CT may represent a highly promising opportunity for improved staging and the radiotherapeutic management of GI tumors in general. Larger, prospective studies will have to confirm these early results.

### 4.3. Pancreas and Liver Cancer

FAP expression is elevated in a pancreatic ductal adenocarcinoma (PDAC) with immunohistochemistry showing FAP expression in >90% of PDACs [103]. As with many other malignancies, FAP expression correlates with the prognosis [103,104]. Thus, it is reasonable to assume that FAPI-PET/CT may be helpful in diagnosing PDAC, which is often difficult with conventional modalities. In a series of 19 patients with PDAC undergoing ^68^Ga-FAPI-PET/CT, all patients demonstrated a high uptake and 10 patients were restaged by FAPI imaging [104]. FAPI-PET/CT may be a better method of facilitating the radiotherapeutic management of PDACs based on its more accurate depiction of the tumor extent. FAPI-PET/CT also showed an improved target volume definition and consistency compared with contrast-enhanced CT [105].

Similarly, FAPI-PET/CT may be of benefit in the early detection of hepatocellular carcinomas. A pilot study assessed 25 patients with hepatic lesions suspicious for a neoplasm. Favorable results for FAPI-PET/CT were achieved with a more accurate identification of malignancy, particularly in earlier diseases [106]. Subsequently, the same group prospectively evaluated the diagnostic performance of FAPI PET/CT in comparison with FDG PET/CT in 20 patients with HCC, finding the former to be more sensitive [107]. These findings were confirmed in a larger study with 34 patients indicating a similar sensitivity of FAPI, ceCT and liver MRI but clear superiority over FDG [49]. Thus, FAPI imaging may enhance the staging, detect a local relapse and guide an accurate treatment in HCC; findings that require additional study for confirmation.

### 4.4. Lymphoma

A prospective study comprising 73 patients with non-Hodgkin and Hodgkin lymphoma investigated the utility of FAPI-PET/CT. Impressive SUV values were documented and a positive correlation was established between FAP expression by immunohistochemistry and the uptake on FAPI PET/CT. Thus, FAPI-PET/CT may find a use in analyzing lymphomas [108].

### 4.5. Sarcoma

FAP is expressed in both the stroma and on neoplastic cells in sarcomas and thus appears important to sarcoma development [42,109,110]. Thus, FAPI-PET/CT might represent a viable imaging and possible therapeutic tool for sarcomas [111,112]. The prospective observational trial by Kessler et al. even established a correlation between a cancerous FAPI accumulation and a histopathological FAP expression as well as a rather high positive predictive value and sensitivity of FAPI-PET/CT [111]. Additional research is required to realize the full potential of FAP expression and FAPI-PET/CT in sarcomas.

### 4.6. Gynecological Malignancies

FAP overexpression has been identified in breast cancer, in particular in the stroma (4) but also in the cancer cells themselves [113]. However, its influence on the prognosis and progression is disputable and inconsistent throughout the literature as several studies suggest a correlation between elevated FAP levels and a poor outcome [114,115] whereas others suggest an association with an improved prognosis [116]. According to first investigations, ^68^Ga-FAPI-PET/CT seems to be highly promising regarding breast cancer. In a prospective pilot study, Kömek et al. analyzed 20 patients with primary or recurrent breast cancer and their metastases and subsequently compared the ^68^Ga-FAPI-PET/CT sensitivity and specificity with those of ^18^F-FDG. FAPI imaging had significantly higher SUV metrics and higher TBRs compared with FDG with the exception of liver lesions, which demonstrated no significant differences [117]. FAPI seems to be of a high interest in several gynecological malignancies as well. An investigation of various malignancies including breast cancer (*n* = 14), ovarian cancer (*n* = 9), cervical cancer (*n* = 4), endometrial cancer (*n* = 2), leiomyosarcoma of the uterus (*n* = 1) and tubal cancer (*n* = 1) described impressive SUV metrics and TBRs and was equal or superior to FDG. Furthermore, immunohistochemistry staining demonstrated a strong FAP expression in ovarian cancer, breast cancer and leiomyosarcoma of the uterus, which is in concordance with previous studies [74]. With respect to ovarian cancer, FAPI-PET/CT might be of particular interest as preceding studies determined its presence in 97% of all ovarian cancers and showed a correlation between FAP and a poor clinical prognosis, chemotherapy resistance and shorter time until recurrence [4,118,119,120,121,122]. In terms of ovarian cancer, the uptake of FAPI-PET/CT was intense [74]. In addition, due to the limited therapeutic options for ovarian cancer, FAPI offers a potential therapeutic target as FAP expression is absent in healthy ovaries [120]. Research on this topic is urgently needed in order to potentially establish a novel diagnostic and therapeutic approach for patients with ovarian cancer. In summary, several gynecological malignancies express FAP and therefore FAPI PET/CT may provide useful diagnostic and eventually theranostic applications. The potential of FAPI-PET/CT in gynecological tumors demands further studies.

### 4.7. FAPI vs. FDG

**Figure 6:**
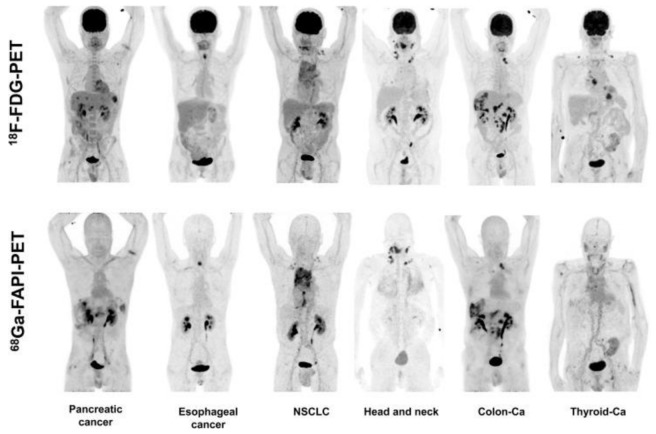
Visualization of Maximum Intensity Projections of intra-indivudal comparisons of FAPI-PET/CT and FDG-PET/CT. Adapted with permission from ref. [128].

^18^F-FDG-PET/CT is the centerpiece of oncological imaging in nuclear medicine. FDG is a glucose-based tracer with enrichment correlating with metabolic activity. However, FDG has several well-known limitations. Fundamentally, it is not tumor-specific. Moreover, FDG has a considerable uptake in several normal tissues such as the brain, liver and bowel. FDG-PET/CT is also of limited use in several low metabolism tumors such as prostate cancer. FAPI-PET/CT could therefore emerge as a supplement or replacement for FDG-PET/CT in a few instances. In large studies of multiple tumor types, FAPI-PET/CT demonstrated a superior diagnostic efficacy [123,124], a more accurate detection [125] and lower background activity [126]. Hence, FAPI may one day “overthrow” FDG from its oncological throne [127] (Figure 6).

## 5. Conclusions

Since the first introduction of quinoline-based FAP ligands in 2018 [5,129,130,131], promising strides have been achieved to enable the improved knowledge of FAP in the field of PET imaging. To date, the precise mechanisms and overexpression of FAP in several malignant and benign diseases remain obscure. FAPI-PET/CT indisputably represents a unique opportunity for diagnostic and therapeutic approaches to tumors and several benign conditions. Further research will be important for establishing the true value of FAPI-PET/CT for patients suffering from cancer and other diseases.

## Data Availability

Data can be asked for based on a reasonable request.

## References

[1] Gascard P., Tlsty T.D. (2016). Carcinoma-associated fibroblasts: Orchestrating the composition of malignancy. Genes Dev..

[2] Barbazán J., Vignjevic D.M. (2018). Cancer associated fibroblasts: Is the force the path to the dark side?. Curr. Opin. Cell Biol..

[3] Hamson E.J., Keane F.M., Tholen S., Schilling O., Gorrell M.D. (2014). Understanding fibroblast activation protein (FAP): Substrates, activities, expression and targeting for cancer therapy. Proteom.—Clin. Appl..

[4] Garin-Chesa P., Old L.J., Rettig W.J. (1990). Cell surface glycoprotein of reactive stromal fibroblasts as a potential antibody target in human epithelial cancers. Proc. Natl. Acad. Sci. USA.

[5] Loktev A., Lindner T., Mier W., Debus J., Altmann A., Jäger D., Giesel F., Kratochwil C., Barthe P., Roumestand C. (2018). A Tumor-Imaging Method Targeting Cancer-Associated Fibroblasts. J. Nucl. Med..

[6] Zi F., He J., He D., Li Y., Yang L., Cai Z. (2015). Fibroblast activation protein α in tumor microenvironment: Recent progression and implications (Review). Mol. Med. Rep..

[7] Scott A.M., Wiseman G., Welt S., Adjei A., Lee F.-T., Hopkins W., Divgi C.R., Hanson L.H., Mitchell P., Gansen D.N. (2003). A Phase I dose-escalation study of sibrotuzumab in patients with advanced or metastatic fibroblast activation protein-positive cancer. Clin. Cancer Res..

[8] Shiga K., Hara M., Nagasaki T., Sato T., Takahashi H., Takeyama H. (2015). Cancer-Associated Fibroblasts: Their Characteristics and Their Roles in Tumor Growth. Cancers.

[9] Gabbiani G., Ryan G.B., Majne G. (1971). Presence of modified fibroblasts in granulation tissue and their possible role in wound contraction. Experientia.

[10] Micallef L., Vedrenne N., Billet F., Coulomb B., Darby I., Desmoulière A. (2012). The myofibroblast, multiple origins for major roles in normal and pathological tissue repair. Fibrogenesis Tissue Repair.

[11] Desmouliere A., Darby I.A., Gabbiani G. (2003). Normal and pathologic soft tissue remodeling: Role of the myofi-broblast, with special emphasis on liver and kidney fibrosis. Lab. Investig..

[12] Desmouliere A., Darby I., Laverdet B., Bonté F. (2014). Fibroblasts and myofibroblasts in wound healing. Clin. Cosmet. Investig. Dermatol..

[13] Muller G.A., Rodemann H.P. (1991). Characterization of human renal fibroblasts in health and disease: I. Immuno-phenotyping of cultured tubular epithelial cells and fibroblasts derived from kidneys with histologically proven interstitial fibrosis. Am. J. Kidney Dis..

[14] Tomasek J.J., Gabbiani G., Hinz B., Chaponnier C., Brown R.A. (2002). Myofibroblasts and mechano-regulation of connective tissue remodelling. Nat. Rev. Mol. Cell Biol..

[15] Sriram G., Bigliardi P.L., Bigliardi-Qi M. (2015). Fibroblast heterogeneity and its implications for engineering or-ganotypic skin m models in vitro. Eur. J. Cell Biol..

[16] Driskell R., Watt F.M. (2015). Understanding fibroblast heterogeneity in the skin. Trends Cell Biol..

[17] Öhlund D., Elyada E., Tuveson D. (2014). Fibroblast heterogeneity in the cancer wound. J. Exp. Med..

[18] Dvorak H.F. (1986). Tumors: Wounds that do not heal. Similarities between tumor stroma generation and wound healing. N. Engl. J. Med..

[19] Driskell R., Lichtenberger B.M., Hoste E., Kretzschmar K., Simons B., Charalambous M., Ferron S., Herault Y., Pavlovic G., Ferguson-Smith A. (2013). Distinct fibroblast lineages determine dermal architecture in skin development and repair. Nature.

[20] Dulauroy S., di Carlo S., Langa F., Eberl G., Peduto L. (2012). Lineage tracing and genetic ablation of ADAM12+ perivascular cells identify a major source of profibrotic cells during acute tissue injury. Nat. Med..

[21] Hamburg-Shields E., DiNuoscio G.J., Mullin N.K., Lafyatis R., Atit R.P., Hamburg E., Lafayatis R. (2014). Sustained β-catenin activity in dermal fibroblasts promotes fibrosis by up-regulating expression of extracellular matrix protein-coding genes. J. Pathol..

[22] Rock J.R., Barkauskas C.E., Cronce M., Xue Y., Harris J.R., Liang J., Noble P.W., Hogan B.L.M. (2011). Multiple stromal populations contribute to pulmonary fibrosis without evidence for epithelial to mesenchymal transition. Proc. Natl. Acad. Sci. USA.

[23] Kalluri R. (2016). The biology and function of fibroblasts in cancer. Nat. Rev. Cancer.

[24] Hwang R.F., Moore T., Arumugam T., Ramachandran V., Amos K.D., Rivera A., Ji B., Evans D.B., Logsdon C.D. (2008). Cancer-Associated Stromal Fibroblasts Promote Pancreatic Tumor Progression. Cancer Res..

[25] Omary M.B., Lugea A., Lowe A.W., Pandol S.J. (2007). The pancreatic stellate cell: A star on the rise in pancreatic diseases. J. Clin. Investig..

[26] Barth P.J., Ebrahimsade S., Ramaswamy A., Moll R. (2001). CD34+ fibrocytes in invasive ductal carcinoma, ductal carcinoma in situ, and benign breast lesions. Virchows Arch..

[27] Mishra P.J., Mishra P.J., Humeniuk R., Medina D.J., Alexe G., Mesirov J.P., Ganesan S., Glod J.W., Banerjee D. (2008). Carcinoma-Associated Fibroblast–Like Differentiation of Human Mesenchymal Stem Cells. Cancer Res..

[28] Weber C.E., Kothari A.N., Wai P.Y., Li N.Y., Driver J., Zapf M.A., Franzen C., Gupta G.N., Osipo C., Zlobin A. (2014). Osteopontin mediates an MZF1–TGF-β1-dependent transformation of mesenchymal stem cells into cancer-associated fibroblasts in breast cancer. Oncogene.

[29] Iwano M., Plieth D., Danoff T.M., Xue C., Okada H., Neilson E.G. (2002). Evidence that fibroblasts derive from epithelium during tissue fibrosis. J. Clin. Investig..

[30] Zeisberg E.M., Potenta S., Xie L., Zeisberg M., Kalluri R. (2007). Discovery of Endothelial to Mesenchymal Transition as a Source for Carcinoma-Associated Fibroblasts. Cancer Res..

[31] Jotzu C., Alt E., Welte G., Li J., Hennessy B.T., Devarajan E., Krishnappa S., Pinilla S., Droll L., Song Y.-H. (2011). Adipose tissue derived stem cells differentiate into carcinoma-associated fibroblast-like cells under the influence of tumor derived factors. Cell. Oncol..

[32] Chen X., Song E. (2019). Turning foes to friends: Targeting cancer-associated fibroblasts. Nat. Rev. Drug. Discov..

[33] Koustoulidou S., Hoorens M.W.H., Dalm S.U., Mahajan S., Debets R., Seimbille Y., de Jong M. (2021). Cancer-Associated Fibroblasts as Players in Cancer Development and Progression and Their Role in Targeted Radionuclide Imaging and Therapy. Cancers.

[34] Tarin D., Croft C.B. (1969). Ultrastructural features of wound healing in mouse skin. J. Anat..

[35] Strutz F., Okada H., Lo C.W., Danoff T., Carone R.L., Tomaszewski J.E., Neilson E.G. (1995). Identification and characterization of a fibroblast marker: FSP1. J. Cell Biol..

[36] Quante M., Tu S.P., Tomita H., Gonda T., Wang S.S., Takashi S., Baik G.H., Shibata W., DiPrete B., Betz K.S. (2011). Bone Marrow-Derived Myofibroblasts Contribute to the Mesenchymal Stem Cell Niche and Promote Tumor Growth. Cancer Cell.

[37] Pietras K., Pahler J., Bergers G., Hanahan D. (2008). Functions of Paracrine PDGF Signaling in the Proangiogenic Tumor Stroma Revealed by Pharmacological Targeting. PLoS Med..

[38] Giannoni E., Bianchini F., Masieri L., Serni S., Torre E., Calorini L., Chiarugi P. (2010). Reciprocal Activation of Prostate Cancer Cells and Cancer-Associated Fibroblasts Stimulates Epithelial-Mesenchymal Transition and Cancer Stemness. Cancer Res..

[39] Aertgeerts K., Levin I., Shi L., Snell G.P., Jennings A., Prasad G.S., Zhang Y., Kraus M.L., Salakian S., Sridhar V. (2005). Structural and Kinetic Analysis of the Substrate Specificity of Human Fibroblast Activation Protein α. J. Biol. Chem..

[40] Sun S., Albright C.F., Fish B.H., George H.J., Selling B.H., Hollis G.F., Wynn R. (2002). Expression, Purification, and Kinetic Characterization of Full-Length Human Fibroblast Activation Protein. Protein Expr. Purif..

[41] Niedermeyer J., Garin-Chesa P., Kriz M., Hilberg F., Mueller E., Bamberger U., Rettig W.J., Schnapp A. (2001). Expression of the fibroblast activation protein during mouse embryo development. Int. J. Dev. Biol..

[42] Rettig W.J., Garin-Chesa P., Beresford H.R., Oettgen H.F., Melamed M.R., Old L.J. (1988). Cell-surface glycoproteins of human sarcomas: Differential expression in normal and malignant tissues and cultured cells. Proc. Natl. Acad. Sci. USA.

[43] Levy M.T., Mccaughan G., Abbott C., Park J.E., Cunningham A.M., Müller E., Rettig W.J., Gorrell M. (1999). Fibroblast activation protein: A cell surface dipeptidyl peptidase and gelatinase expressed by stellate cells at the tissue remodelling interface in human cirrhosis. Hepatology.

[44] Bauer S., Jendro M.C., Wadle A., Kleber S., Stenner F., Dinser R., Reich A., Faccin E., Gödde S., Dinges H. (2006). Fibroblast activation protein is expressed by rheumatoid myofibroblast-like synoviocytes. Arthritis Res..

[45] Brokopp C.E., Schoenauer R., Richards P., Bauer S., Lohmann C., Emmert M.Y., Weber B., Winnik S., Aikawa E., Graves K. (2011). Fibroblast activation protein is induced by inflammation and degrades type I collagen in thin-cap fibroatheromata. Eur. Heart J..

[46] Fitzgerald A.A., Weiner L.M. (2020). The role of fibroblast activation protein in health and malignancy. Cancer Metastasis Rev..

[47] De Willige S.U., Malfliet J.J.M.C., Janssen H.L.A., Leebeek F.W.G., Rijken D.C. (2013). Increased N-terminal cleavage of alpha-2-antiplasmin in patients with liver cirrhosis. J. Thromb. Haemost..

[48] Zhao L., Gu J., Fu K., Lin Q., Chen H. (2020). 68Ga-FAPI PET/CT in Assessment of Liver Nodules in a Cirrhotic Patient. Clin. Nucl. Med..

[49] Guo W., Pang Y., Yao L., Zhao L., Fan C., Ke J., Guo P., Hao B., Fu H., Xie C. (2020). Imaging fibroblast activation protein in liver cancer: A single-center post hoc retrospective analysis to compare [68Ga] Ga-FAPI-04 PET/CT versus MRI and [18F]-FDG PET/CT. Eur. J. Nucl. Med. Mol. Imaging.

[50] Rovedatti L., Knowles C.H., Sengupta N., Corazza G.R., Di Sabatino A., Biancheri P., Macdonald T.T. (2011). Fibroblast activation protein expression in Crohn’s disease strictures. Inflamm. Bowel Dis..

[51] Zidar N., Langner C., Jerala M., Boštjančič E., Drobne D., Tomažič A. (2020). Pathology of Fibrosis in Crohn’s Disease—Contribution to Understanding Its Pathogenesis. Front. Med..

[52] Luo Y., Pan Q., Xu H., Zhang R., Li J., Li F. (2020). Active uptake of 68Ga-FAPI in Crohn’s disease but not in ulcerative colitis. Eur. J. Nucl. Med. Mol. Imaging.

[53] Wasserman A. (2018). Rheumatoid Arthritis: Common Questions About Diagnosis and Management. Am. Fam. Physician.

[54] Glyn-Jones S., Palmer A.J.R., Agricola R., Price A.J., Vincent T., Weinans H., Carr A.J. (2015). Osteoarthritis. Lancet.

[55] Milner J.M., Kevorkian L., Young D.A., Jones D., Wait R., Donell S.T., Barksby E., Patterson A.M., Middleton J., Cravatt B.F. (2006). Fibroblast activation protein alpha is expressed by chondrocytes following a pro-inflammatory stimulus and is elevated in osteoarthritis. Arthritis Res..

[56] Xu T., Zhao Y., Ding H., Cai L., Zhou Z., Song Z., Chen Y. (2020). [68Ga] Ga-DOTA-FAPI-04 PET/CT imaging in a case of prostate cancer with shoulder arthritis. Eur. J. Nucl. Med. Mol. Imaging.

[57] Luo Y., Pan Q., Yang H., Li F., Zhang F. (2021). Inflammatory Arthritis Induced by Anti-Programmed Death-1 Shown in 68Ga-FAPI PET/CT in a Patient with Esophageal Carcinoma. Clin. Nucl. Med..

[58] Tillmanns J., Widera C., Habbaba Y., Galuppo P., Kempf T., Wollert K.C., Bauersachs J. (2013). Circulating concentrations of fibroblast activation protein α in apparently healthy individuals and patients with acute coronary syndrome as assessed by sandwich ELISA. Int. J. Cardiol..

[59] De Willige S.U., Malfliet J.J., Deckers J.W., Dippel D.W., Leebeek F.W., Rijken D.C. (2015). Plasma levels of soluble fibroblast activation protein in arterial thrombosis; determinants and cleavage of its substrate alpha-2-antiplasmin. Int. J. Cardiol..

[60] Tillmanns J., Hoffmann D., Habbaba Y., Schmitto J., Sedding D., Fraccarollo D., Galuppo P., Bauersachs J. (2015). Fibroblast activation protein alpha expression identifies activated fibroblasts after myocardial infarction. J. Mol. Cell. Cardiol..

[61] Varasteh Z., Mohanta S., Robu S., Braeuer M., Li Y., Omidvari N., Topping G., Sun T., Nekolla S.G., Richter A. (2019). Molecular Imaging of Fibroblast Activity After Myocardial Infarction Using a 68Ga-Labeled Fibroblast Activation Protein Inhibitor, FAPI-04. J. Nucl. Med..

[62] Notohamiprodjo S., Nekolla S.G., Robu S., Asiares A.V., Kupatt C., Ibrahim T., Laugwitz K.-L., Makowski M.R., Schwaiger M., Weber W.A. (2021). Imaging of cardiac fibroblast activation in a patient after acute myocardial infarction using 68Ga-FAPI. J. Nucl. Cardiol..

[63] Totzeck M., Siebermair J., Rassaf T., Rischpler C. (2019). Cardiac fibroblast activation detected by positron emission tomography/computed tomography as a possible sign of cardiotoxicity. Eur. Heart J..

[64] Heckmann M.B., Reinhardt F., Finke D., Katus H.A., Haberkorn U., Leuschner F., Lehmann L.H. (2020). Relationship Between Cardiac Fibroblast Activation Protein Activity by Positron Emission Tomography and Cardiovascular Disease. Circ. Cardiovasc. Imaging.

[65] Siebermair J., Köhler M.I., Kupusovic J., Nekolla S.G., Kessler L., Ferdinandus J., Guberina N., Stuschke M., Grafe H., Siveke J.T. (2020). Cardiac fibroblast activation detected by Ga-68 FAPI PET imaging as a potential novel biomarker of cardiac injury/remodeling. J. Nucl. Cardiol..

[66] Finke D., Heckmann M.B., Herpel E., Katus H.A., Haberkorn U., Leuschner F., Lehmann L.H. (2021). Early Detection of Checkpoint Inhibitor-Associated Myocarditis Using 68Ga-FAPI PET/CT. Front. Cardiovasc. Med..

[67] Luo Y., Pan Q., Yang H., Peng L., Zhang W., Li F. (2020). Fibroblast Activation Protein–Targeted PET/CT with 68Ga-FAPI for Imaging IgG4-Related Disease: Comparison to 18F-FDG PET/CT. J. Nucl. Med..

[68] Schmidkonz C., Rauber S., Atzinger A., Agarwal R., Götz T.I., Soare A., Cordes M., Prante O., Bergmann C., Kleyer A. (2020). Disentangling inflammatory from fibrotic disease activity by fibroblast activation protein imaging. Ann. Rheum. Dis..

[69] Zhang X., Song W., Qin C., Liu F., Lan X. (2021). Non-malignant findings of focal 68Ga-FAPI-04 uptake in pancreas. Eur. J. Nucl. Med. Mol. Imaging.

[70] Röhrich M., Leitz D., Glatting F.M., Wefers A.K., Weinheimer O., Flechsig P., Kahn N., Mall M.A., Giesel F.L., Kratochwil C. (2021). Fibroblast Activation Protein specific PET/CT imaging in fibrotic interstitial lung diseases and lung cancer: A translational exploratory study. J. Nucl. Med..

[71] Qin C., Gai Y., Liu Q., Shao F., Lan X. (2020). Elevated 68Ga-FAPI Accumulation in a Recurrent Angiomyolipoma. Clin. Nucl. Med..

[72] Liu H., Liu L., Chen L., Zhao Y., Zhang W., Cai L., Chen Y. (2021). [68Ga] Ga-DOTA-FAPI-04 PET/CT imaging of benign pulmonary solitary fibrous tumour. Eur. J. Nucl. Med. Mol. Imaging.

[73] Zheng S., Lin R., Chen S., Zheng J., Lin Z., Zhang Y., Xue Q., Chen Y., Zhang J., Lin K. (2021). Characterization of the benign lesions with increased 68Ga-FAPI-04 uptake in PET/CT. Ann. Nucl. Med..

[74] Dendl K., Koerber S.A., Finck R., Mokoala K.M.G., Staudinger F., Schillings L., Heger U., Röhrich M., Kratochwil C., Sathekge M. (2021). 68Ga-FAPI-PET/CT in patients with various gynecological malignancies. Eur. J. Nucl. Med. Mol. Imaging.

[75] Dendl K., Koerber S.A., Adeberg S., Röhrich M., Kratochwil C., Haberkorn U., Giesel F.L. (2021). Physiological FAP-activation in a postpartum woman observed in oncological FAPI-PET/CT. Eur. J. Nucl. Med. Mol. Imaging.

[76] Sonni I., Lee-Felker S., Memarzadeh S., Quinn M.M., Mona C.E., Lückerath K., Czernin J., Calais J. (2020). 68Ga-FAPi-46 diffuse bilateral breast uptake in a patient with cervical cancer after hormonal stimulation. Eur. J. Nucl. Med. Mol. Imaging.

[77] Wang L.-J., Zhang Y., Wu H.-B. (2020). Intense Diffuse Uptake of 68Ga-FAPI-04 in the Breasts Found by PET/CT in a Patient with Advanced Nasopharyngeal Carcinoma. Clin. Nucl. Med..

[78] Dendl K., Schlittenhardt J., Staudinger F., Kratochwil C., Altmann A., Haberkorn U., Giesel F.L. (2021). The Role of Fibroblast Activation Protein Ligands in Oncologic PET Imaging. PET Clin..

[79] Kratochwil C., Flechsig P., Lindner T., Abderrahim L., Altmann A., Mier W., Adeberg S., Rathke H., Röhrich M., Winter H. (2019). 68Ga-FAPI PET/CT: Tracer Uptake in 28 Different Kinds of Cancer. J. Nucl. Med..

[80] Dendl K., Finck R., Giesel F.L., Kratochwil C., Lindner T., Mier W., Cardinale J., Kesch C., Röhrich M., Rathke H. (2021). FAP imaging in rare cancer entities—first clinical experience in a broad spectrum of malignancies. Eur. J. Nucl. Med. Mol. Imaging.

[81] Stremenova J., Krepela E., Mares V., Trim J., Dbaly V., Marek J., Vanickova Z., Lisa V., Yea C., Sedo A. (2007). Expression and enzymatic activity of dipeptidyl peptidase-IV in human astrocytic tumours are associated with tumour grade. Int. J. Oncol..

[82] Matrasova I., Busek P., Balaziova E., Sedo A. (2017). Heterogeneity of molecular forms of dipeptidyl pep-tidase-IV and fibroblast activation protein in human glioblastomas. Biomed Pap..

[83] Mentlein R., Hattermann K., Hemion C., Jungbluth A.A., Held-Feindt J. (2011). Expression and role of the cell surface protease seprase/fibroblast activation protein-α (FAP-α) in astroglial tumors. Biol. Chem..

[84] Busek P., Balaziova E., Matrasova I., Hilser M., Tomas R., Syrucek M., Zemanova Z., Krepela E., Belacek J., Sedo A. (2016). Fibroblast activation protein alpha is expressed by transformed and stromal cells and is associated with mesenchymal features in glioblastoma. Tumor Biol..

[85] Röhrich M., Loktev A., Wefers A.K., Altmann A., Paech D., Adeberg S., Windisch P., Hielscher T., Flechsig P., Floca R. (2019). IDH-wildtype glioblastomas and grade III/IV IDH-mutant gliomas show elevated tracer uptake in fibroblast activation protein–specific PET/CT. Eur. J. Nucl. Med. Mol. Imaging.

[86] Windisch P., Röhrich M., Regnery S., Tonndorf-Martini E., Held T., Lang K., Bernhardt D., Rieken S., Giesel F., Haberkorn U. (2020). Fibroblast Activation Protein (FAP) specific PET for advanced target volume delineation in glioblastoma. Radiother. Oncol..

[87] Syed M., Flechsig P., Liermann J., Windisch P., Staudinger F., Akbaba S., Koerber S.A., Freudlsperger C., Plinkert P.K., Debus J. (2020). Fibroblast activation protein inhibitor (FAPI) PET for diagnostics and advanced targeted radiotherapy in head and neck cancers. Eur. J. Nucl. Med. Mol. Imaging.

[88] Linz C., Brands R.C., Kertels O., Dierks A., Brumberg J., Gerhard-Hartmann E., Hartmann S., Schirbel A., Serfling S., Zhi Y. (2021). Targeting fibroblast activation protein in newly diagnosed squamous cell carcinoma of the oral cavity—initial experience and comparison to [18F] FDG PET/CT and MRI. Eur. J. Nucl. Med. Mol. Imaging.

[89] Röhrich M., Syed M., Liew D.P., Giesel F.L., Liermann J., Choyke P.L., Wefers A.K., Ritz T., Szymbara M., Schillings L. (2021). 68Ga-FAPI-PET/CT improves diagnostic staging and radiotherapy planning of adenoid cystic carcinomas—Imaging analysis and histological validation. Radiother. Oncol..

[90] Okada K., Chen W.-T., Iwasa S., Jin X., Yamane T., Ooi A., Mitsumata M. (2003). Seprase, a Membrane-Type Serine Protease, Has Different Expression Patterns in Intestinal- and Diffuse-Type Gastric Cancer. Oncology.

[91] Mori Y., Kono K., Matsumoto Y., Fujii H., Yamane T., Mitsumata M., Chen W.-T. (2004). The Expression of a Type II Transmembrane Serine Protease (Seprase) in Human Gastric Carcinoma. Oncology.

[92] Hu M., Qian C., Hu Z., Fei B., Zhou H. (2017). Biomarkers in Tumor Microenvironment? Upregulation of Fibroblast Activation Protein-α Correlates with Gastric Cancer Progression and Poor Prognosis. OMICS A J. Integr. Biol..

[93] Wen X., He X., Jiao F., Wang C., Sun Y., Ren X., Li Q. (2017). Fibroblast Activation Protein-α-Positive Fibroblasts Promote Gastric Cancer Progression and Resistance to Immune Checkpoint Blockade. Oncol. Res. Featur. Preclin. Clin. Cancer Ther..

[94] Pang Y., Zhao L., Luo Z., Hao B., Wu H., Lin Q., Sun L., Chen H. (2021). Comparison of 68Ga-FAPI and 18F-FDG Uptake in Gastric, Duodenal, and Colorectal Cancers. Radiology.

[95] Qin C., Shao F., Gai Y., Liu Q., Ruan W., Liu F., Hu F., Lan X. (2021). 68Ga-DOTA-FAPI-04 PET/MR in the evaluation of gastric carcinomas: Comparison with 18F-FDG PET/CT. J. Nucl. Med..

[96] Zhao L., Pang Y., Luo Z., Fu K., Yang T., Zhao L., Sun L., Wu H., Lin Q., Chen H. (2021). Role of [68Ga] Ga-DOTA-FAPI-04 PET/CT in the evaluation of peritoneal carcinomatosis and comparison with [18F]-FDG PET/CT. Eur. J. Nucl. Med. Mol. Imaging.

[97] Ristau J., Giesel F.L., Haefner M.F., Staudinger F., Lindner T., Merkel A., Schlittenhardt J., Kratochwil C., Choyke P.L., Herfarth K. (2020). Impact of Primary Staging with Fibroblast Activation Protein Specific Enzyme Inhibitor (FAPI)-PET/CT on Radio-Oncologic Treatment Planning of Patients with Esophageal Cancer. Mol. Imaging Biol..

[98] Zhao L., Chen S., Chen S., Pang Y., Dai Y., Hu S., Lin L., Fu L., Sun L., Wu H. (2021). 68Ga-fibroblast activation protein inhibitor PET/CT on gross tumour volume delineation for radiotherapy planning of oesophageal cancer. Radiother. Oncol..

[99] Iwasa S., Jin X., Okada K., Mitsumata M., Ooi A. (2003). Increased expression of seprase, a membrane-type serine protease, is associated with lymph node metastasis in human colorectal cancer. Cancer Lett..

[100] Henry L.R., Lee H.-O., Lee J.S., Klein-Szanto A., Watts P., Ross E.A., Chen W.-T., Cheng J.D. (2007). Clinical Implications of Fibroblast Activation Protein in Patients with Colon Cancer. Clin. Cancer Res..

[101] Wikberg M.L., Edin S., Lundberg I.V., Van Guelpen B., Dahlin A.M., Rutegård J., Stenling R., Öberg Å., Palmqvist R. (2013). High intratumoral expression of fibroblast activation protein (FAP) in colon cancer is associated with poorer patient prognosis. Tumor Biol..

[102] Koerber S.A., Staudinger F., Kratochwil C., Adeberg S., Haefner M.F., Ungerechts G., Rathke H., Winter E., Lindner T., Syed M. (2020). The Role of 68Ga-FAPI PET/CT for Patients with Malignancies of the Lower Gastrointestinal Tract: First Clinical Experience. J. Nucl. Med..

[103] Shi M., Yu D.-H., Chen Y., Zhao C.-Y., Zhang J., Liu Q.-H., Ni C.-R., Zhu M.-H. (2012). Expression of fibroblast activation protein in human pancreatic adenocarcinoma and its clinicopathological significance. World J. Gastroenterol..

[104] Lo A., Li C.-P., Buza E.L., Blomberg R., Govindaraju P., Avery D., Monslow J., Hsiao M., Puré E. (2017). Fibroblast activation protein augments progression and metastasis of pancreatic ductal adenocarcinoma. JCI Insight.

[105] Liermann J., Syed M., Ben-Josef E., Schubert K., Schlampp I., Sprengel S., Ristau J., Weykamp F., Röhrich M., Koerber S. (2021). Impact of FAPI-PET/CT on Target Volume Definition in Radiation Therapy of Locally Recurrent Pancreatic Cancer. Cancers.

[106] Shi X., Xing H., Yang X., Li F., Yao S., Zhang H., Zhao H., Hacker M., Huo L., Li X. (2020). Fibroblast imaging of hepatic carcinoma with 68Ga-FAPI-04 PET/CT: A pilot study in patients with suspected hepatic nodules. Eur. J. Nucl. Med. Mol. Imaging.

[107] Shi X., Xing H., Yang X., Li F., Yao S., Congwei J., Zhao H., Hacker M., Huo L., Li X. (2020). Comparison of PET imaging of activated fibroblasts and 18F-FDG for diagnosis of primary hepatic tumours: A prospective pilot study. Eur. J. Nucl. Med. Mol. Imaging.

[108] Jin X., Wei M., Wang S., Wang G., Lai Y., Shi Y., Zhang Y., Yang Z., Wang X. (2021). Detecting fibroblast activation proteins in lymphoma using 68Ga-FAPI PET/CT. J. Nucl. Med..

[109] Dohi O., Ohtani H., Hatori M., Sato E., Hosaka M., Nagura H., Itoi E., Kokubun S. (2009). Histogenesis-specific expression of fibroblast activation protein and dipeptidylpeptidase-IV in human bone and soft tissue tumours. Histopathology.

[110] Ding L., Ye L., Xu J., Jiang W.G. (2014). Impact of fibroblast activation protein on osteosarcoma cell lines in vitro. Oncol. Lett..

[111] Kessler L., Ferdinandus J., Hirmas N., Bauer S., Dirksen U., Zarrad F., Nader M., Chodyla M.-K., Milosevic A., Umutlu L. (2021). Ga-68-FAPI as diagnostic tool in sarcoma: Data from the FAPI-PET prospective observational trial. J. Nucl. Med..

[112] Koerber S.A., Finck R., Dendl K., Uhl M., Lindner T., Kratochwil C., Röhrich M., Rathke H., Ungerechts G., Adeberg S. (2021). Novel FAP ligands enable improved imaging contrast in sarcoma patients due to FAPI-PET/CT. Eur. J. Nucl. Med. Mol. Imaging.

[113] Goodman J.D., Rozypal T.L., Kelly T. (2003). Seprase, a membrane-bound protease, alleviates the serum growth requirement of human breast cancer cells. Clin. Exp. Metastasis.

[114] Yu H., Yang J., Li Y., Jiao S. (2015). Xi bao yu fen zi mian yi xue za zhi. Chin. J. Cell. Mol. Immunol..

[115] Jia J., Martin T.A., Ye L., Jiang W.G. (2014). FAP-α (Fibroblast activation protein-α) is involved in the control of human breast cancer cell line growth and motility via the FAK pathway. BMC Cell Biol..

[116] Ariga N., Sato E., Ohuchi N., Nagura H., Ohtani H. (2001). Stromal expression of fibroblast activation protein/seprase, a cell membrane serine proteinase and gelatinase, is associated with longer survival in patients with invasive ductal carcinoma of breast. Int. J. Cancer.

[117] Kömek H., Can C., Güzel Y., Oruç Z., Gündoğan C., Yildirim A., Kaplan I., Erdur E., Yıldırım M.S., Çakabay B. (2021). 68Ga-FAPI-04 PET/CT, a new step in breast cancer imaging: A comparative pilot study with the 18F-FDG PET/CT. Ann. Nucl. Med..

[118] Jin X., Iwasa S., Okada K., Mitsumata M., Ooi A. (2003). Expression patterns of seprase, a membrane serine protease, in cervical carcinoma and cervical intraepithelial neoplasm. Anticancer Res..

[119] Lai N., Ma L., Wang F. (2012). Fibroblast activation protein regulates tumor-associated fibroblasts and epithelial ovarian cancer cells. Int. J. Oncol..

[120] Hussain A., Voisin V., Poon S., Karamboulas C., Bui N.H.B., Meens J., Dmytryshyn J., Ho V.W., Tang K.H., Paterson J. (2020). Distinct fibroblast functional states drive clinical outcomes in ovarian cancer and are regulated by TCF21. J. Exp. Med..

[121] Mhawech-Fauceglia P., Yan L., Sharifian M., Ren X., Liu S., Kim G., Gayther S.A., Pejovic T., Lawrenson K. (2014). Stromal Expression of Fibroblast Activation Protein Alpha (FAP) Predicts Platinum Resistance and Shorter Recurrence in patients with Epithelial Ovarian Cancer. Cancer Microenviron..

[122] Zhang Y., Tang H., Cai J., Zhang T., Guo J., Feng D., Wang Z. (2011). Ovarian cancer-associated fibroblasts contribute to epithelial ovarian carcinoma metastasis by promoting angiogenesis, lymphangiogenesis and tumor cell invasion. Cancer Lett..

[123] Chen H., Pang Y., Wu J., Zhao L., Hao B., Wu J., Wei J., Wu S., Zhao L., Luo Z. (2020). Comparison of [68Ga] Ga-DOTA-FAPI-04 and [18F] FDG PET/CT for the diagnosis of primary and metastatic lesions in patients with various types of cancer. Eur. J. Nucl. Med. Mol. Imaging.

[124] Chen H., Zhao L., Ruan D., Pang Y., Hao B., Dai Y., Wu X., Guo W., Fan C., Wu J. (2020). Usefulness of [68Ga] Ga-DOTA-FAPI-04 PET/CT in patients presenting with inconclusive [18F] FDG PET/CT findings. Eur. J. Nucl. Med. Mol. Imaging.

[125] Ballal S., Yadav M.P., Moon E.S., Kramer V.S., Roesch F., Kumari S., Tripathi M., ArunRaj S.T., Sarswat S., Bal C. (2020). Biodistribution, pharmacokinetics, dosimetry of [68Ga] Ga-DOTA.SA.FAPi, and the head-to-head comparison with [18F] F-FDG PET/CT in patients with various cancers. Eur. J. Nucl. Med. Mol. Imaging.

[126] Giesel F.L., Kratochwil C., Schlittenhardt J., Dendl K., Eiber M., Staudinger F., Kessler L., Fendler W.P., Lindner T., Koerber S.A. (2021). Head-to-head intra-individual comparison of biodistribution and tumor uptake of 68Ga-FAPI and 18F-FDG PET/CT in cancer patients. Eur. J. Nucl. Med. Mol. Imaging.

[127] Calais J., Mona C.E. (2021). Will FAPI PET/CT Replace FDG PET/CT in the Next Decade? Point—An Important Diagnostic, Phenotypic, and Biomarker Role. Am. J. Roentgenol..

[128] Giesel F.L., Kratochwil C., Lindner T., Marschalek M.M., Loktev A., Lehnert W., Debus J., Jäger D., Flechsig P., Altmann A. (2019). ^68^Ga-FAPI PET/CT: Biodistribution and Preliminary Dosimetry Estimate of 2 DOTA-Containing FAP-Targeting Agents in Patients with Various Cancers. J. Nucl. Med..

[129] Lindner T., Loktev A., Altmann A., Giesel F., Kratochwil C., Debus J., Jäger D., Mier W., Haberkorn U. (2018). Development of Quinoline-Based Theranostic Ligands for the Targeting of Fibroblast Activation Protein. J. Nucl. Med..

[130] Loktev A., Lindner T., Burger E.-M., Altmann A., Giesel F., Kratochwil C., Debus J., Marme F., Jäger D., Mier W. (2019). Development of Fibroblast Activation Protein-Targeted Radiotracers with Improved Tumor Retention. J. Nucl. Med..

[131] Lindner T., Altmann A., Kraemer S., Kleist C., Loktev A., Kratochwil C., Giesel F., Mier W., Marme F., Debus J. (2020). Design and Development of 99mTc-Labeled FAPI Tracers for SPECT Imaging and 188Re Therapy. J. Nucl. Med..

